# Supplementary material for: Predicting mortality among patients with liver cirrhosis in electronic health records with machine learning
Source: PLoS One. 2021 Aug 31;16(8):e0256428. doi: 10.1371/journal.pone.0256428 (PMC8407576; doi:10.1371/journal.pone.0256428)
Supplement: S1 Fig — Figure a, b, c is for the case of mortality within 365 days, 180 days, and 90 days, respectively. (PDF) [file pone.0256428.s001.pdf]

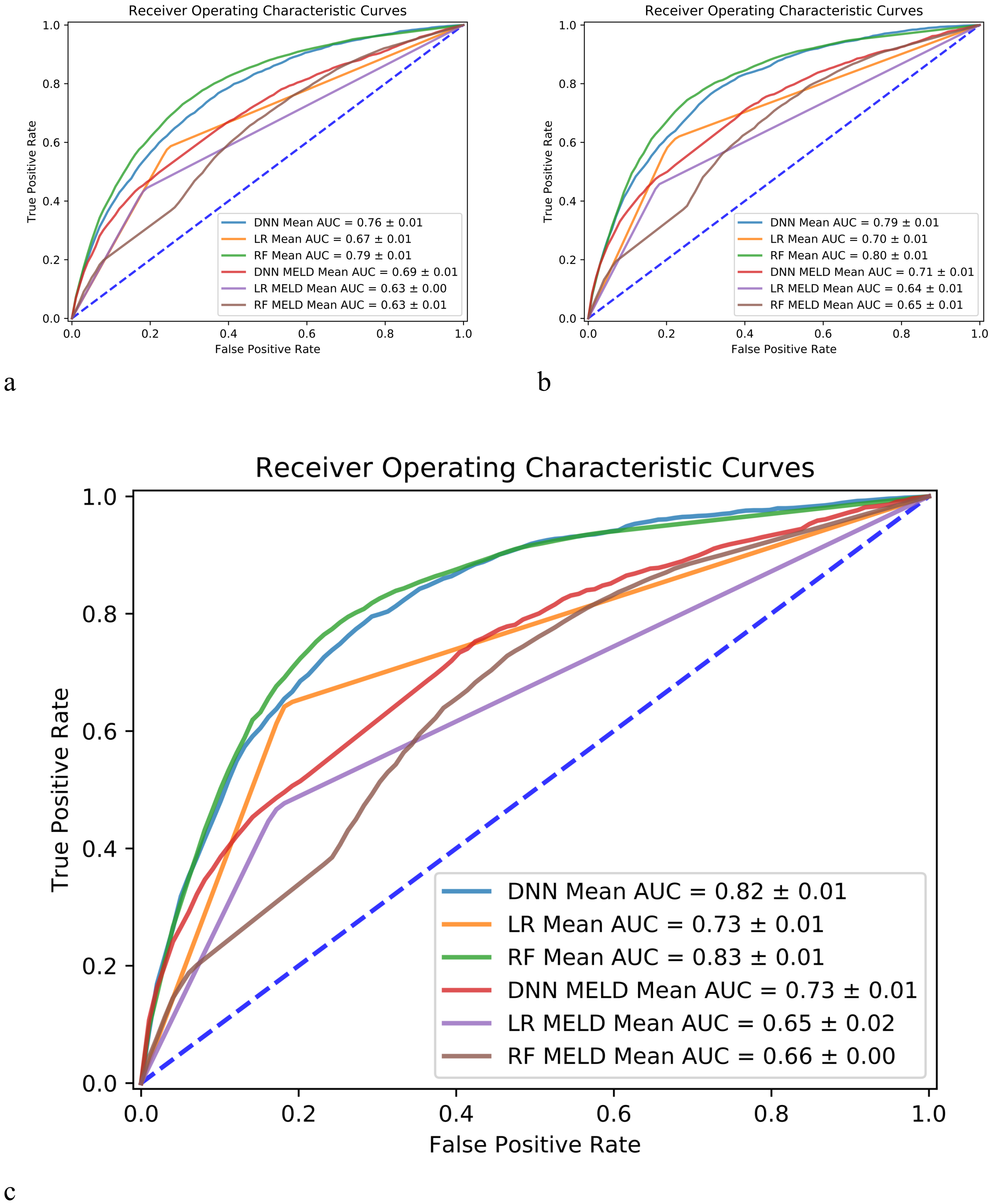

S1 Fig. Prediction performance by deep neural network (DNN), random forest (RF) and logistic regression (LR) with mean imputation strategy. Figure a, b, c is for the case of mortality within 365 days, 180 days, and 90 days, respectively.
