# Supplementary material for: Predicting mortality among patients with liver cirrhosis in electronic health records with machine learning
Source: PLoS One. 2021 Aug 31;16(8):e0256428. doi: 10.1371/journal.pone.0256428 (PMC8407576; doi:10.1371/journal.pone.0256428)
Supplement: S1 Table — (DOCX) [file pone.0256428.s002.docx]

**S1 Table.** The study features and feature description.

| Supplemental Table S1 | |
| --- | --- |
| Feature names | Feature description and value examples |
| Gender | Gender (e.g., Female, Male) |
| Primary race | Race (e.g., White, Black, Asian, Other) |
| Ethnicity | Ethnicity (e.g., Hispanic or Latino, Not Hispanic or Latino) |
| Age at event | Age of patients when the first time diagnosed with HF |
| Associated visit type | Visit types (e.g., Inpatient visit, Outpatient visit, Emergency room visit, Observation \\ Same day Visit, Ancillary, Pre-visit, Series) |
| Condition | Condition types (Unspecified cirrhosis of liver, Alcoholic cirrhosis of liver without ascites, Biliary cirrhosis, Other specified disorders of liver, Esophageal varices without bleeding, Esophageal varices without mention of bleeding, Hereditary hemochromatosis, Other cirrhosis of liver, Portal hypertension, Primary biliary cirrhosis, Alcoholic cirrhosis of liver with ascites, Gastric varices, Chronic passive congestion of liver, Viral hepatitis A with hepatic coma, Hepatorenal syndrome, Spontaneous bacterial peritonitis, Unspecified viral hepatitis C with hepatic coma, Wilson's disease, 18 Esophageal varices with bleeding, Hepatopulmonary syndrome) |
| Condition type | Condition types (e.g., Diagnosis, Problem) |
| Present on admission | If cirrhosis present on admission (e.g., Yes, No, Ns) |
| Diagnosis type | Diagnosis types (e.g, Reason For Visit, Final, Final Diagnosis, Admitting) |
| Ascites-Condition | Ascites condition (e.g., Alcoholic hepatitis with ascites) |
| hosp-Admission start date | Days between cirrhosis diagnosis dates and hospital (hosp) admission start date |
| hosp-Admission end date | Days between cirrhosis diagnosis dates and hospital admission end date |
| bmi-Average calculated bmi | The numerical value of body mass index (bmi) |
| bmi-Average weight | The numerical value of weight |
| bmi-Average height | The numerical value of height |
| smk-Alcohol use | If use alcohol (e.g., Yes, No); smk - smoking |
| Reference Event-Facility | Facility (e.g., BJC/Washington University) |
| sodium-Result value numeric | The numeric value of sodium |
| sodium-Age at event | Age of patients at the measure of INR |
| INR-Result value numeric | The numeric value of INR |
| INR-Age at event | Age of patients at the measure of INR |
| creatinine-Result value numeric | The numeric value of creatinine |
| creatinine-Age at event | Age of patients at the measure of creatinine |
| Tbili-Result value numeric | The numeric value of Tbili |
| Tbili-Age at event | Age of patients at the measure of Tbili |
| Mcv-Estimated result | The numeric value of Mcv |
| Mcv-Age at event | Age of patients at the measure of Mcv |
| hemoglobin-Result value numeric | The numeric value of hemoglobin |
| hemoglobin-Age at event | Age of patients at the measure of hemoglobin |
| potassium-Result value numeric | The numeric value of potassium |
| potassium-Age at event | Age of patients at the measure of potassium |
| bicarbonate-Result value numeric | The numeric value of bicarbonate |
| bicarbonate-Age at event | Age of patients at the measure of bicarbonate |
| alt-Result value numeric | The numeric value of alt (Alanine aminotransferase) |
| alt-Age at event | Age of patients at the measure of alt |
| ast-Result value numeric | The numeric value of ast (Aspartate) |
| ast-Age at event | Age of patients at the measure of ast |
| alkaline-Result value numeric | The numeric value of alkaline |
| alkaline-Age at event | Age of patients at the measure of alkaline |
| abo/Rh-Age at event | Age of patients at the measure of blood group |
| AFP-Age at event | Age of patients at the measure of AFP(Alpha-1-Fetoprotein [Presence]) |
