# Supplementary material for: Predicting mortality among patients with liver cirrhosis in electronic health records with machine learning
Source: PLoS One. 2021 Aug 31;16(8):e0256428. doi: 10.1371/journal.pone.0256428 (PMC8407576; doi:10.1371/journal.pone.0256428)
Supplement: S2 Table — (DOCX) [file pone.0256428.s003.docx]

**S2 Table. Prediction Metrics [n (%)] of 3 period cases for 3 machine learning models with mean strategy for imputation.**

| Models | Period  (days) | Accuracy  Mean(std) | Precision  Mean(std) | Recall  Mean(std) | F1-Score  Mean(std) | Specificity  Mean(std) |
| --- | --- | --- | --- | --- | --- | --- |
| DNN  (all variables) | 365 | 0.81(0.01) | 0.21(0.01) | 0.50(0.03) | 0.30(0.01) | 0.84(0.03) |
|  | 180 | 0.83(0.01) | 0.19(0.0) | 0.54 (0.03) | 0.28(0.01) | 0.85(0.03) |
|  | 90 | 0.85(0.01) | 0.18(0.0) | 0.58(0.02) | 0.28(0.00) | 0.86(0.01) |
| LR  (all variables) | 365 | 0.74(0.0) | 0.17(0.0) | 0.58(0.02) | 0.26(0.01) | 0.75 (0.01) |
|  | 180 | 0.78(0.01) | 0.16(0.01) | 0.61(0.02) | 0.26(0.01) | 0.79(0.01) |
|  | 90 | 0.81 (0.01) | 0.16(0.01) | 0.64(0.02) | 0.26(0.01) | 0.82 (0.01) |
| RF  (all variables) | 365 | 0.89 (0.0) | 0.30(0.01) | 0.32(0.02) | 0.31(0.01) | 0.94 (0.0) |
|  | 180 | 0.90 (0.0) | 0.26(0.01) | 0.31(0.02) | 0.28(0.02) | 0.94 (0.0) |
|  | 90 | 0.91 (0.0) | 0.25(0.02) | 0.33(0.03) | 0.28(0.02) | 0.95(0.0) |
| DNN  (4 MELD-Na variables) | 365 | 0.82(0.02) | 0.20(0.02) | 0.39(0.03) | 0.26(0.02) | 0.86(0.03) |
|  | 180 | 0.80(0.0) | 0.15(0.0) | 0.45(0.02) | 0.22(0.01) | 0.82(0.0) |
|  | 90 | 0.77(0.09) | 0.12(0.02) | 0.52(0.12) | 0.20(0.02) | 0.79(0.10) |
| LR  (4 MELD-Na variables) | 365 | 0.79(0.01) | 0.17(0.01) | 0.44(0.0) | 0.25(0.01) | 0.82(0.01) |
|  | 180 | 0.77(0.01) | 0.12(0.00) | 0.42(0.02) | 0.19(0.01) | 0.79(0.01) |
|  | 90 | 0.81(0.01) | 0.13(0.01) | 0.47(0.04) | 0.20(0.02) | 0.83(0.01) |
| RF  (4 MELD-Na variables) | 365 | 0.83(0.07) | 0.16(0.03) | 0.22(0.07) | 0.18(0.01) | 0.89(0.08) |
|  | 180 | 0.76(0.07) | 0.11(0.04) | 0.34(0.08) | 0.15(0.02) | 0.78(0.08) |
|  | 90 | 0.73(0.0) | 0.08 (0.0) | 0.39(0.03) | 0.13(0.01) | 0.75(0.0) |
