# Supplementary material for: Predicting mortality among patients with liver cirrhosis in electronic health records with machine learning
Source: PLoS One. 2021 Aug 31;16(8):e0256428. doi: 10.1371/journal.pone.0256428 (PMC8407576; doi:10.1371/journal.pone.0256428)
Supplement: S3 Table — (DOCX) [file pone.0256428.s004.docx]

**S3 Table. Prediction Metrics [n(%)] of 3 machine learning models under 10 different tradeoffs for case of 365 days.**

| Models | Tradeoff | Accuracy  Mean(std) | Precision  Mean(std) | Recall  Mean(std) | F1-Score  Mean(std) | Specificity  Mean(std) |
| --- | --- | --- | --- | --- | --- | --- |
| DNN | 0.05 | 0.52 (0.06) | 0.14 (0.01) | 0.93 (0.02) | 0.24 (0.02) | 0.48 (0.07) |
|  | 0.1 | 0.63 (0.06) | 0.17 (0.02) | 0.87 (0.04) | 0.28 (0.02) | 0.61 (0.07) |
|  | 0.2 | 0.78 (0.01) | 0.22 (0.01) | 0.71 (0.01) | 0.34 (0.01) | 0.79 (0.01) |
|  | 0.3 | 0.79 (0.02) | 0.22 (0.01) | 0.69 (0.05) | 0.34 (0.02) | 0.79 (0.03) |
|  | 0.4 | 0.82 (0.03) | 0.25 (0.03) | 0.62 (0.03) | 0.35 (0.03) | 0.83 (0.04) |
|  | 0.6 | 0.86 (0.01) | 0.3 (0.02) | 0.51 (0.03) | 0.37 (0.02) | 0.9 (0.01) |
|  | 0.7 | 0.88 (0.01) | 0.31 (0.02) | 0.44 (0.02) | 0.37 (0.02) | 0.92 (0.01) |
|  | 0.8 | 0.91 (0.0) | 0.41 (0.03) | 0.33 (0.04) | 0.37 (0.03) | 0.96 (0.0) |
|  | 0.9 | 0.93 (0.0) | 0.6 (0.06) | 0.21 (0.02) | 0.31 (0.02) | 0.99 (0.0) |
|  | 0.95 | 0.93 (0.0) | 0.86 (0.04) | 0.16 (0.03) | 0.26 (0.04) | 1.0 (0.0) |
| LR | 0.05 | 0.25 (0.01) | 0.09 (0.0) | 0.96 (0.0) | 0.17 (0.0) | 0.19 (0.01) |
|  | 0.1 | 0.35 (0.01) | 0.1 (0.0) | 0.92 (0.0) | 0.19 (0.0) | 0.3 (0.01) |
|  | 0.2 | 0.51 (0.01) | 0.12 (0.0) | 0.85 (0.01) | 0.22 (0.0) | 0.48 (0.01) |
|  | 0.3 | 0.61 (0.01) | 0.14 (0.0) | 0.78 (0.01) | 0.24 (0.0) | 0.6 (0.01) |
|  | 0.4 | 0.7 (0.01) | 0.17 (0.01) | 0.71 (0.02) | 0.27 (0.01) | 0.7 (0.01) |
|  | 0.6 | 0.82 (0.01) | 0.23 (0.01) | 0.53 (0.02) | 0.32 (0.01) | 0.85 (0.01) |
|  | 0.7 | 0.86 (0.0) | 0.27 (0.01) | 0.44 (0.02) | 0.33 (0.01) | 0.9 (0.0) |
|  | 0.8 | 0.89 (0.0) | 0.32 (0.01) | 0.32 (0.01) | 0.32 (0.01) | 0.94 (0.0) |
|  | 0.9 | 0.91 (0.0) | 0.37 (0.04) | 0.15 (0.02) | 0.22 (0.03) | 0.98 (0.0) |
|  | 0.95 | 0.92 (0.0) | 0.39 (0.07) | 0.07 (0.01) | 0.12 (0.02) | 0.99 (0.0) |
| RF | 0.05 | 0.51 (0.01) | 0.13 (0.0) | 0.93 (0.01) | 0.23 (0.0) | 0.48 (0.01) |
|  | 0.1 | 0.63 (0.01) | 0.16 (0.0) | 0.88 (0.01) | 0.28 (0.0) | 0.61 (0.01) |
|  | 0.2 | 0.77 (0.01) | 0.22 (0.01) | 0.75 (0.01) | 0.34 (0.01) | 0.77 (0.01) |
|  | 0.3 | 0.85 (0.0) | 0.28 (0.01) | 0.62 (0.01) | 0.39 (0.01) | 0.86 (0.01) |
|  | 0.4 | 0.89 (0.0) | 0.36 (0.01) | 0.5 (0.01) | 0.42 (0.01) | 0.92 (0.0) |
|  | 0.6 | 0.92 (0.0) | 0.49 (0.02) | 0.29 (0.02) | 0.36 (0.02) | 0.97 (0.0) |
|  | 0.7 | 0.92 (0.0) | 0.53 (0.03) | 0.21 (0.01) | 0.3 (0.02) | 0.98 (0.0) |
|  | 0.8 | 0.93 (0.0) | 0.62 (0.03) | 0.15 (0.01) | 0.25 (0.01) | 0.99 (0.0) |
|  | 0.9 | 0.93 (0.0) | 0.73 (0.04) | 0.11 (0.01) | 0.19 (0.01) | 1.0 (0.0) |
|  | 0.95 | 0.93 (0.0) | 0.82 (0.04) | 0.09 (0.01) | 0.16 (0.01) | 1.0 (0.0) |
