# Supplementary material for: Predicting mortality among patients with liver cirrhosis in electronic health records with machine learning
Source: PLoS One. 2021 Aug 31;16(8):e0256428. doi: 10.1371/journal.pone.0256428 (PMC8407576; doi:10.1371/journal.pone.0256428)
Supplement: Attachment — Submitted filename: Response to Reviewers.docx [file pone.0256428.s006.docx]

**Predicting mortality among patients with liver cirrhosis in electronic health records with machine learning**

*We would like to thank the reviewers for their informed, thoughtful, and helpful comments. Please find our responses to the reviews below in italics. We hope that they will find it suitable for publication in the PlOS ONE.*

**Review Comments to the Author

Please use the space provided to explain your answers to the questions above. You may also include additional comments for the author, including concerns about dual publication, research ethics, or publication ethics. (Please upload your review as an attachment if it exceeds 20,000 characters)**

**Reviewer #1: (No Response)**

**Reviewer #2: The authors have largely addressed my concerns. I still question the use of features with only more than 10% non-missing values. My understanding this is contrary to common practice which would require a much higher proportion of non-missing values.**

*We appreciate and agree with the reviewer’s comment, and our apologies for the confusion in our original manuscript and the response of the first-round revision. Among all these 41 features, most of the features had low missing value rates (<20%), and only 4 features had high missing value rate (shown in the following Table 1). We tried to keep more features/variables for the study.*

**Table 1. Missing value rates of all studied 41 features.**

| Variables | Non-missing value rate (%) | Missing value rate (%) |
| --- | --- | --- |
| Gender | 100 | 0 |
| Primary race | 97.36 | 2.64 |
| Ethnicity | 67.84 | 32.16 |
| Age at event | 100 | 0 |
| Associated visit type | 86.43 | 13.57 |
| Condition | 100 | 0 |
| Condition type | 100 | 0 |
| Present on admission | 84.22 | 15.78 |
| Diagnosis type | 84.22 | 15.78 |
| Ascites-Condition | 100 | 0 |
| hosp-Admission start date | 65.42 | 34.58 |
| hosp-Admission end date | 65.42 | 34.58 |
| bmi-Average calculated bmi | 55.49 | 44.51 |
| bmi-Average weight | 61.45 | 38.55 |
| bmi-Average height | 58 | 42 |
| smk-Alcohol use | 48.13 | 51.87 |
| Reference Event-Facility | 99.99 | 0.01 |
| sodium-Result value numeric | 80.49 | 19.51 |
| sodium-Age at event | 80.49 | 19.51 |
| INR-Result value numeric | 62.08 | 37.92 |
| INR-Age at event | 62.15 | 37.85 |
| creatinine-Result value numeric | 79.99 | 20.01 |
| creatinine-Age at event | 80.06 | 19.94 |
| Tbili-Result value numeric | 77.01 | 22.99 |
| Tbili-Age at event | 77.39 | 22.61 |
| Mcv-Estimated result | 79.87 | 20.13 |
| Mcv-Age at event | 79.87 | 20.13 |
| hemoglobin-Result value numeric | 80.9 | 19.1 |
| hemoglobin-Age at event | 80.9 | 19.1 |
| potassium-Result value numeric | 80.56 | 19.44 |
| potassium-Age at event | 80.57 | 19.43 |
| bicarbonate-Result value numeric | 16.19 | 83.81 |
| bicarbonate-Age at event | 16.19 | 83.81 |
| alt-Result value numeric | 76.55 | 23.45 |
| alt-Age at event | 76.71 | 23.29 |
| ast-Result value numeric | 77.42 | 22.58 |
| ast-Age at event | 77.42 | 22.58 |
| alkaline-Result value numeric | 77.37 | 22.63 |
| alkaline-Age at event | 77.39 | 22.61 |
| abo/Rh-Age at event | 22.62 | 77.38 |
| AFP-Age at event | 14.84 | 85.16 |
